# Genetic and Clinical Characterization of Danish Achromatopsia Patients

**DOI:** 10.3390/genes14030690

**Published:** 2023-03-10

**Authors:** Mette Kjøbæk Gundestrup Andersen, Mette Bertelsen, Karen Grønskov, Susanne Kohl, Line Kessel

**Affiliations:** 1 Department of Ophthalmology, Copenhagen University Hospital—Rigshospitalet, 2600 Glostrup, Denmark; 2 Department of Clinical Genetics, Copenhagen University Hospital—Rigshospitalet, 2100 Copenhagen, Denmark; 3 Institute for Ophthalmic Research, Center for Ophthalmology, University of Tübingen, 72076 Tübingen, Germany; 4 Department of Clinical Medicine, University of Copenhagen, 2200 Copenhagen, Denmark

**Keywords:** Achromatopsia, *CNGA3*, *CNGB3*, *GNAT2*, *PDE6C*, *PDE6H*

## Abstract

Achromatopsia is a rare congenital condition with cone photoreceptor dysfunction causing color blindness, reduced vision, nystagmus and photophobia. New treatments are being developed, but the current evidence is still conflicting regarding possible progression over time, and there is no clear genotype-phenotype correlation. This natural history study aimed to further explore the course of disease and potential clinical differences between various genotypes. The retrospective design allowed for the study of a large cohort with a long follow-up. Patients were identified from the Danish national registries. If not already available, genetic analysis was offered to the patient. Clinical data from 1945–2022 were retrieved from medical records and included best-corrected visual acuity (BCVA), color vision, refractive error, nystagmus, visual fields and fundoscopic findings. We identified variants believed to be disease causing in five of the known achromatopsia genes: *CNGA3*; *CNGB3*; *GNAT2*; *PDE6C* and *PDE6H*; and novel variants were identified in *CNGB3* and *PDE6C*. Progressive deterioration of BCVA only attributable to achromatopsia was found in three of 58 patients. Progressive phenotype was seen with variants in *CNGB3* and *PDE6C.* The results indicate that myopia could be more frequently occurring with variants in *GNAT2*, *PDE6C* and *PDE6H* and support the evidence that achromatopsia is a predominantly stationary condition with respect to BCVA. Although a clear genotype-phenotype correlation can still not be concluded, there may be differences in phenotypical characteristics with variants in different genes.

## 1. Introduction

Achromatopsia (ACHM) is a rare congenital, autosomal recessively inherited retinal condition with an estimated prevalence of 1 in 30,000–50,000. Since it is caused by dysfunction of cone photoreceptors, it is also known as rod monochromacy. Gene therapy trials are ongoing and have shown promising results [1,2], but uncertainties about the natural history of the condition calls for natural history studies with a long follow-up.

Clinically, ACHM is characterized by an inability to see colors, low visual acuity of around 20/200, photo-aversion and nystagmus. Complete ACHM is the most common form, but incomplete ACHM with a milder phenotype has also been described [3]. Fundoscopically, the retina is often unremarkable or has subtle abnormalities, such as central mottling or an absent foveolar reflex, but central atrophy can also be seen [4]. The condition is often diagnosed in infancy or early childhood based on typical clinical findings and demonstration of a lack of cone function by electroretinography [5]. Changes in the outer retinal layers, such as disruption in the inner segment ellipsoid layer or outer retinal atrophy, may be seen on optic coherence tomography (OCT) [6].

Achromatopsia has classically been described as a stationary condition, but some studies have found that the severity of certain phenotypic characteristics correspond with increasing age, indicating progression in some patients. These characteristics include the loss of cone inner and outer segments on OCT [7], macular atrophy [4] and poorer visual acuity [8]. Other studies have not found this correlation [6,9,10]. The severity/characteristics seen on OCT and fundoscopy do not necessarily correspond to the severity of symptoms experienced by the patient [6]. More severe phenotypes of progressive cone and cone-rod dystrophy have also been described with variants in the genes typically causing ACHM [11]. The association between genotypes and phenotypes, both in terms of residual cone function and disease progression, remains unclear [12].

The molecular basis for ACHM is most often found in cone phototransduction since five of the six identified genes involved in ACHM encode proteins involved in this cascade: *CNGA3* (MIM#216900) [13], *CNGB3* (MIM#262300) [14], *GNAT2* (MIM#613856) [15], *PDE6C* (MIM#600827) [16,17], and *PDE6H* (MIM#601190) [18]. The sixth and most recently identified gene is *ATF6* (MIM#616517), which encodes an activating transcription factor essential for cone photoreceptor development [19,20,21]. Gene therapy trials delivering *CNGA3* and *CNGB3* have been completed [1,2] (NCT02610582, NTC03758404) or are ongoing (NCT02935517, NCT03278873, NCT02599922, NCT03001310), but further knowledge on the potential progressive nature of the disorder would be useful in establishing the most optimal time to deliver a possible future treatment, as would further exploration of genotype-phenotype associations and the identification of new, potentially pathogenic variants. In this study, we aimed to identify the genetic spectrum of Danish patients with ACHM; provide clinical data from medical records, thus enabling a natural history study with a long follow-up; and explore possible genotype-phenotype associations.

## 2. Materials and Methods

We aimed to include all Danish patients with genetically verified ACHM in the study. Patients were identified from the Danish Family Archive for Genetic Eye Disease, the Danish Register for the Blind and Partially Sighted Children and electronic medical charts. Briefly, these registries hold information about Danish patients with hereditary eye diseases and visually impaired and blind children. The registries are located at the National Eye Clinic for the Visually Impaired at the Kennedy Center, which also has medical records dating back more than a hundred years.

Patients with a clinical diagnosis of ACHM, cone dysfunction and/or variants in one of the known ACHM genes were identified. If genetic analysis was not already available, or previous results needed confirmation, and the patient was still alive, he/she was contacted and offered genetic testing to confirm the diagnosis. Only patients in whom the diagnosis could be genetically confirmed were included.

Blood samples from patients were collected from 1999 to 2021. Before 2017, DNA was mainly analyzed by Sanger sequencing of coding regions and adjacent intronic sequences of known ACHM genes. After 2017, genetic analysis of ACHM genes was performed using customized SureSelect libraries (Agilent, Santa Clara, CA, USA), followed by next-generation sequencing (NGS) using Illumina technology (San Diego, CA, USA) and a MiSeq machine. Genes associated with ACHM were included in a larger panel of genes associated with inherited retinal disease. Alignment and variant calling were performed using SureCall (Agilent) and interpretation using VarSeq (Golden Helix, Bozeman, MT, USA) and Alamut (Lausanne, Switzerland). For some patients the genetic diagnosis was already published in previous studies [15,22,23,24,25,26,27,28]. All variants were classified according to the American College of Medical Genetics and Genomics (ACMG) guidelines [29] and additional general recommendations from the ClinGen working group (https://clinicalgenome.org/working-groups/sequence-variant-interpretation/, accessed on 6 January 2023). Achromatopsia was considered genetically confirmed if two pathogenic or likely pathogenic variants in one of the six known genes (*CNGA3*, *CNGB3*, *GNAT2*, *PDE6C*, *PDE6H*, *ATF6*) were identified in the patient or an affected sibling. In a few cases (four patients, two variants) one pathogenic or likely pathogenic variant was identified together with a variant classified as a variant of unknown significance (VUS). These cases were still included since the genetic findings overall were believed to be a likely cause fitting with the phenotype. It was not possible to confirm that the variants were in trans for all patients because parental samples were not always available.

Clinical data from 1945 to 2022 were retrieved and recorded. They included best-corrected visual acuity (BCVA), refractive error, color vision, visual fields, history of childhood nystagmus and fundoscopic findings. Results of electroretinography exams were not included in this study since different test modalities have been used over the years, and ISCEV standards were only introduced in recent years. Optical coherence tomography scans (OCT) were not consistently available for review. For a few patients some clinical findings have been published in previous studies, either along with genetic findings [22,24,27,28,30] or before genetic analyses were available [31,32,33].

Best-corrected visual acuity measurement was converted from Snellen to logMAR for analysis. For small children and patients with developmental delays, visual acuity was measured by symbol recognition (Østerberg or Kay acuity charts). Measurements based on preferential looking were not evaluated in the current study since they were not precise enough to compare with later measurements using symbol recognition. To evaluate changes in BCVA over time, the last available measurement was compared to the first measurement. Only data from patients older than 9 years old were analyzed since these patients were able to cooperate with Snellen visual acuity measurement with a reliable result. A change in BCVA of ≥0.2 logMAR was considered clinically significant [34].

The latest refractive data were noted. Cycloplegic autorefraction was preferred for patients younger than 30 years of age and non-mydriatic autorefraction for patients older than 30 years old, but alternatively, the prescribed refractive correction was noted if autorefraction measurements were not available. Spherical equivalent refractive error (SER = spherical refractive error + ½ cylinder) was calculated and analyzed. Myopia was defined as SER ≤ −0.5 diopters, hyperopia as SER ≥ +0.5 diopters and emmetropia as SER < + 0.50 and >−0.50 diopters. Statistical analysis included Fisher’s pairwise exact test comparing refractive data with variants in two different genes for three outcome calculations (myopia, emmetropia and hyperopia). Calculations were performed using R statistical software, version 4.2.2 (R Project for Statistical Computing, Vienna, Austria).

Color vision tests were reviewed. Throughout the years, different tests have been used and included Ishihara pseudoisochromatic plates, Farnsworth–Munsell (FM) 15 or 28 saturated and/or Nagel anomaloscopy. Complete colorblindness was concluded if the patient was only able to see the first Ishihara plate, had multiple diagonal errors (>3) on FM and/or achromatic color matching on anomaloscopy. Some patients were tested using more than one modality. In these cases, the reliability of the test was considered. In accordance with previous definitions [3,35], anomaloscopy was deemed the most reliable test in establishing complete colorblindness and Ishihara the least reliable.

A mild phenotype consistent with incomplete achromatopsia was recorded if the patient had a combination of BCVA < 1.0 logMAR and residual color vision.

Fundoscopic findings were reviewed, and it was recorded if there was central chorioretinal atrophy at the latest follow-up. There was some variation in the description of the fundoscopic findings from visit to visit and between different clinicians, especially if findings were subtle, but the presence/absence of central atrophy was a consistent description. When available, fundus photographs were reviewed to confirm the findings reported in the medical record. Goldman visual fields were reviewed when available. If these measurements were not available, descriptions of confrontation visual fields were used.

## 3. Results

We identified 89 patients carrying one apparent homozygous or two heterozygous variants believed to be disease causing in known ACHM genes. Eighteen patients had a diagnosis of ACHM or cone dysfunction but were unavailable for genetic testing and were not included. Both clinical and genetic data were available for 85 patients. The median age at the last follow-up was 31 years old (IQR 33, range 2–76 years). The median follow-up was 17 years (IQR 21, range 0–71 years). Six patients only had clinical data from one visit. In 21 families more than one sibling had ACHM, and eight of these families were consanguineous.

### 3.1. Genetic Characterization

**Figure 1:**
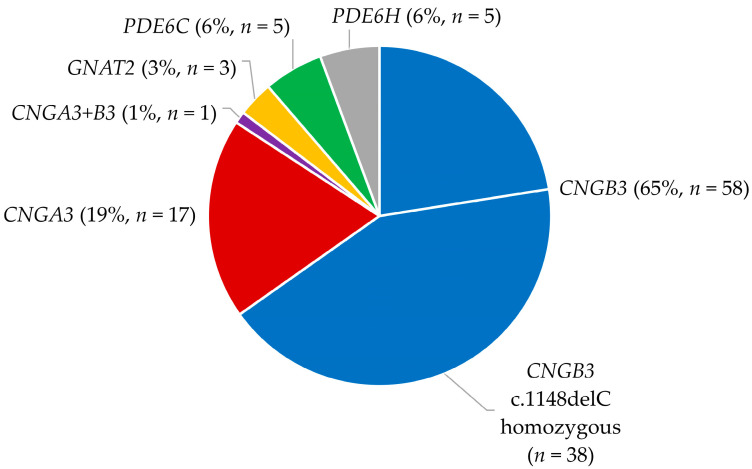
Distribution of genes with disease-causing variants in Danish ACHM patients.

Variants in *CNGB3* were the most common cause of ACHM (65.2% of patients; see Figure 1 and Supplementary Table S1), and 65.5% (38 patients) of these patients were homozygous for the variant c.1148delC causing a frameshift and introduction of a premature stop codon (p.T383Ifs*13). The c.1148delC variant accounted for 50.3% of all mutant alleles in our cohort. In one patient, three pathogenic variants were identified: two in *CNGB3* and one in *CNGA3*. We identified one novel pathogenic variant in *CNGB3*, the other identified variants have been published previously (see Supplementary Table S1) [14,23,25,36,37]. Most variants identified in *CNGB3* led to a premature stop codon, and they were most frequently caused by small deletions.

Variants in *CNGA3* were the second most frequent cause of ACHM in our cohort and were most predominantly missense variants resulting from single base pair substitutions, and have all been previously published [13,24,38,39,40,41,42].

Variants in other genes were less common and included missense and splice site variants in *PDE6C* [17,28]. We identified four new *PDE6C* variants that were classified as pathogenic, one likely pathogenic variant and one VUS. All patients with causative variants in *PDE6H* had a previously published [18] single base pair substitution resulting in a premature stop codon. The variants in *GNAT2* have all been previously published and all resulted in a premature stop codon (see Supplementary Table S1) [15,22,27].

### 3.2. Clinical Characterization

Best-corrected visual acuity at latest follow-up ranged from 0.52 to 1.30 logMAR with a median of 1.00 logMAR (IQR 0.20); see Table 1.

**Figure 2:**
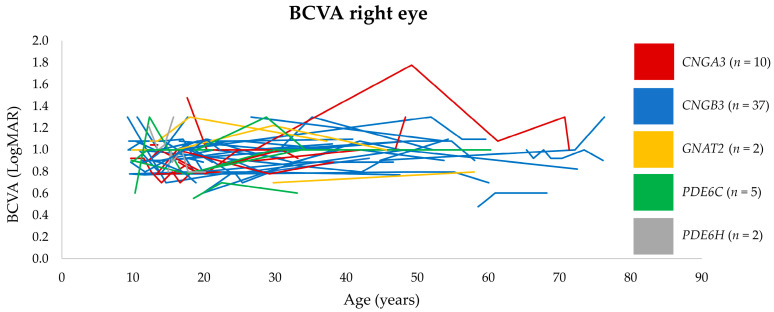
Spaghetti plot of the development in BCVA for 58 ACHM patients. Only data for the right eye are shown.

Fifty-eight patients had at least two BCVA measurements available after the age of 9 years old, enabling longitudinal analysis with a median follow-up of 22 years (IQR 23, range 1–65 years); see Figure 2. Small fluctuations in visual acuity were seen during follow-up, but the first and last measurements were within ±0.2 logMAR in the majority (88%, *n* = 51) of patients. In seven patients, a clinically significant deterioration of more than 0.2 logMAR was observed in one eye (three patients) or both eyes (four patients). In three patients, the visual decline was unrelated to ACHM: cataract (*n* = 2) and corneal ulcer (*n* = 1). In one patient, BCVA deteriorated from 0.8 to 1.0 logMAR from age 11 to 17 years old in one eye, which also had exotropia. In three patients, the visual decline could not be explained by ocular comorbidities; one of these patients had deterioration of BCVA in one eye only.

Color vision testing was performed in 57 patients, 49 of these patients had complete color blindness, and eight had residual color vision (see also Table 1). Five of them also had BCVA better than 1.00 logMAR in both eyes at the latest follow-up, consistent with incomplete ACHM.

Most patients (89%, *n* = 72) had a history of childhood nystagmus, but eight had no history of nystagmus; two of these patients also had the other characteristics of incomplete ACHM.

Three patients (5%) had visual field constriction to <30 degrees. Central scotoma was not recorded in any of the patients.

**Figure 3:**
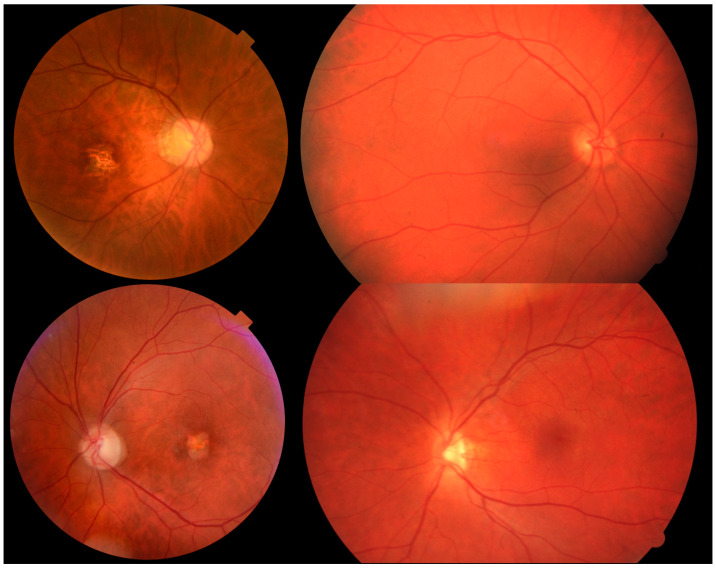
Representative fundus photograph of patients with (left images) and without (right images) central chorioretinal atrophy. The upper panel shows the right eyes of two patients with variants in *CNGA3* (c.1641C > A;p.(F54L) homozygous and c.1574G > A;p.(G525D) and c.1694C > T;p.(T565M) respectively) aged 47 and 57 years old, respectively. The lower panel shows the left eyes of two patients both homozygous for c.1148delC in *CNGB3* aged 68 and 42 years old, respectively.

Fundoscopic findings varied from no pathologies to central chorioretinal atrophy. In 14 patients (19%), chorioretinal atrophy was noted at the latest follow-up, the median age for these patients was 56 years old (IQR 18.25, range 19–72 years), and eight of these patients had BCVA of 1.00 logMAR or better. Representative fundus pictures of patients with variants in *CNGA3* and *CNGB3* with and without central chorioretinal atrophy are shown in Figure 3. The worst BCVA observed with central atrophy was 1.30.

**Figure 4:**
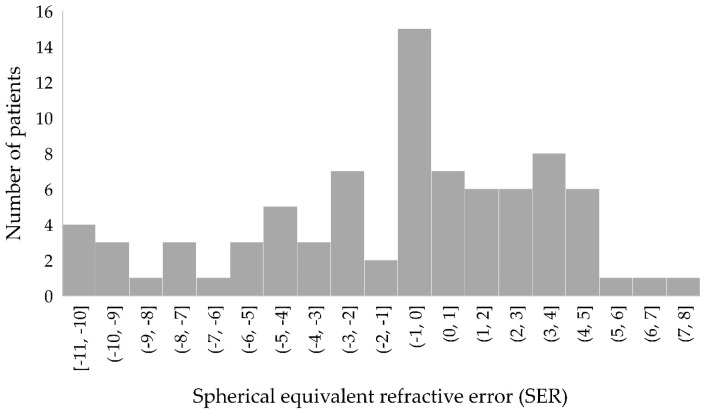
Distribution of the spherical equivalent refractive error (SER) of all patients. Only data for the right eye are shown.

Refractive data were available for 83 patients; see Figure 4. The median spherical equivalent refractive error (SER) was 0.00 diopters for both eyes (IQR 5.88 for right eye and 5.63 for left eye). Forty-nine percent of patients had hyperopia with SER ≥ +0.5 diopters, 34% had myopia with SER ≤ −0.50 diopters, and 17% were emmetropic (SER > −0.50 and <+0.50 diopters). 

### 3.3. Genotype-Phenotype Correlations

Generally, the clinical characteristics were similar in patients with causative variants in the five identified genes, but some differences were observed (for a summary, see Table 1).

Median visual acuity was slightly better for patients with variants in *CNGA3* and *PDE6H*, but a BCVA < 1.0 logMAR was found in patients with variants in all five identified genes. Best corrected visual acuity worse than 1.00 logMAR was not observed in patients with variants in *PDE6H*. Incomplete colorblindness was also seen in cases with variants in all five identified genes. Three patients with residual color vision had variants in *CNGB3*, the genotype of two of these patients was c.1148delC;p.(T383fs) and c.1208G > A;p.(R403Q), and the genotype of the third patient was c.1148delC;p.(T383fs) and c.886–896del11insT;p.(T296fs).

A combination of BCVA < 1.0 logMAR in both eyes and residual color vision at the latest follow up consistent with incomplete ACHM was found in five patients, with variants in four different genes (*CNGA3 n* = 2, *CNGB3 n* = 1, *GNAT2 n* = 1, *PDE6H n* = 1), but not in patients with causative variants in *PDE6C*. Two patients with incomplete ACHM and variants in *CNGA3* (c.1574G > A;p.(G525D) and c.1694C > T;p.(T565M)) were twins. Incomplete and complete ACHM was observed in a sibling pair with variants in *CNGB3* (c.1148delC;p.(T383fs) and c.886–896del11insT;p.(T3296fs)). One patient with residual color vision had BCVA < 1.0 logMAR in the left eye only and deterioration of BCVA in the right (exotropic) eye; the genotype for this patient was *CNGB3* c.1148delC;p.(T383fs) and c.1208G > A;p.(R403Q). One other patient shared this genotype and had residual color vision but demonstrated a clinically significant worsening of BCVA in both eyes during follow-up. Two other patients with causative variants in *CNGB3* and residual color vision and demonstrated clinically significant worsening of BCVA throughout follow-up, but for these patients, the deterioration could also be explained by ocular comorbidities. Deterioration of BCVA was also seen in two patients with variants in *PDE6C*, but in one of them, the deterioration was unrelated to ACHM. Worsening of BCVA was not seen with variants in *GNAT2* and *PDE6H*. Complete ACHM was seen in a single patient with variants in *PDE6H.*

Congenital nystagmus was frequent in all five gene groups (see Table 1).

Visual field constriction <30 degrees was rarely seen (*n* = 3) and only in patients with causative variants in *CNGA3* and *CNGB3*. For the patient with visual field constriction and variants in *CNGA3*, pigmentary changes were noted in the retinal periphery; this patient also had worsening of BCVA, which could also be attributable to cataract. The patients with *CNGB3* variants did not have peripheral retinal changes. One had a pale optic disc noted on fundoscopy; the other had normal fundoscopic findings.

Central atrophy was not observed with causative variants in *GNAT2* and *PDE6H.*

**Figure 5:**
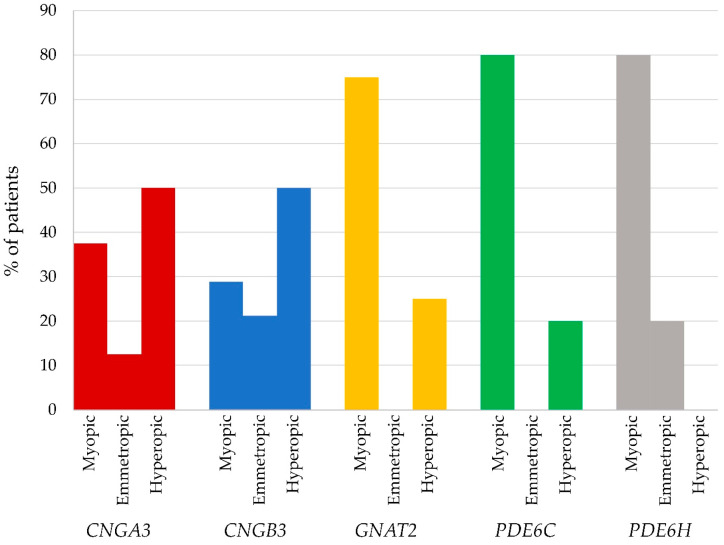
Percentage of patients with different types of refractive error within the groups of different causal genes. Myopia is defined as SER ≤ −0.50 diopters and hyperopia as SER ≥ +0.50 diopters.

Data on refractive errors at latest follow up was available for 82 patients, with variants in the five different genes (*CNGA3 n* = 16, *CNGB3 n* = 52, *GNAT2 n* = 4, *PDE6C n* = 5, *PDE6H n* = 5). Refractive errors varied among the five gene groups (see Figure 5). Patients with variants in *CNGA3* and *CNGB3* exhibited a range of different refractive errors and in both groups 50% were hyperopic ≥ +0.50 diopters, with 12.5% and 21% respectively being emmetropic. Myopia was apparently more frequent in patients with variants in *GNAT2*, *PDE6C* and *PDE6H* (75, 80 and 80%, respectively). Differences in refractive error with variants in different genes using pairwise analyses was statistically significant when comparing patients with variants in *CNGB3* and *PDE6H* (*p*-value 0.028). Severe myopia (SER ≤ −6.00 diopters) was observed with variants in all five genes but was more frequently occurring in patients with variants in *PDE6H* (100%, 4 of 4 patients with myopia), *PDE6C* (75%, 3 of 4 patients) and *GNAT2* (33%, 1 of 3 patients) than in patients with variants in *CNGB3* (20%, 3 of 15 patients) and *CNGA3* (17%, 1 of 6 patients).

## 4. Discussion

In this study we describe the genetic background and natural history of Danish patients with ACHM based on a review of medical records. We present a large cohort consisting of 85 patients and a long follow-up for most patients.

We found BCVA to remain relatively stable over time but clinically significant deterioration attributable to ACHM was found in three of 58 patients (5%). This finding supports the notion that ACHM is a predominantly stationary condition, at least as far as it is experienced by patients. Other studies have found similar results [9,43,44], and a single study also described fluctuations in the measured BCVA [43], as we also found. However, a recent Italian study of patients with variants in *CNGA3*, *CNGB3* and *GNAT2* also described deterioration in BCVA over time in 10 of 16 patients over a mean follow-up of 5.4 years [8], so the evidence is still not conclusive. In this study, we focused on changes in BCVA that would be relevant to the patient and therefore deemed a change in BCVA of >0.2 logMAR to be clinically significant [34] and a sign of progressive disease. However, to our knowledge, no studies exist on which levels of visual acuity deterioration are noticeable to patients with ACHM or other forms of severe congenital visual impairment.

The genetic background of Danish ACHM patients found in this study is overall consistent with that from other studies in Northern Europe, with variants in *CNGB3* being the most frequent cause and c.1148delC being the most frequent disease-causing variant [23]. Variants in *PDE6C* and *PDE6H* were more frequent in this study than the <2% previously documented [15,17,18]. Patients with causative variants in *PDE6H* came from two different consanguineous families originating from Syria and Turkey, so it is unlikely that there is a higher prevalence of *PDE6H* variants in the general Danish population. In contrast, patients with variants in *PDE6C* were unrelated, except for two patients, so it is possible that the prevalence of *PDE6C* variants in Denmark is higher than in other Northern European populations. We did not identify any patients with causative variants in *ATF6*.

In agreement with earlier studies [12,36] we found that *CNGA3* variants were predominantly missense variants, and the majority of variants in *CNGB3* were small deletions. Variants in *GNAT2* all resulted in a premature stop codon. We identified novel variants in *CNGB3* and *PDE6C*. The single base pair substitution identified in *PDE6H* is the only variant associated with ACHM identified in this gene to date [18,45].

Genotype-phenotype correlations are typically sparse in ACHM with regard to severity of the disease [12]. A milder phenotype of incomplete ACHM has been described in patients with variants in *CNGA3*, *CNGB3*, *GNAT2*, *PDE6C* and *PDE6H* [17,18,24,27,46]., and to our knowledge, *PDE6H* is exclusively associated with incomplete ACHM [18]. The Danish patients with incomplete ACHM had variants in *CNGA3*, *CNGB3*, *GNAT2* and *PDE6H* but not in *PDE6C.* Incomplete ACHM was most frequent with variants in *CNGA3*, but data on color vision were only available for the few patients with variants in *PDE6H*. One patient with variants in *PDE6H* had a phenotype consistent with complete ACHM, thereby broadening the phenotypic spectrum for this gene.

Progressive disease has been described in patients with variants in *CNGA3*, *CNGB3*, *GNAT2* and *PDE6C* [17,24,27,37] but infrequently with variants in *GNAT2* and not at all with variants in *PDE6H* [18]. In concordance with these findings, we did not find deterioration of BCVA with variants *GNAT2* and *PDE6H*.

For patients with variants in *CNGA3* and worsening of BCVA, this outcome could also be explained by cataract, so in our study, a progressive phenotype only attributable to ACHM was only seen in patients with variants in *CNGB3* and *PDE6C.* This finding is consistent with previous findings indicating that progressive cone [11,37] and cone-rod dystrophies [47] are more frequently reported for these two genes.

Regarding *CNGB3,* we identified three unrelated patients with the c.1208G > A;p.(R403Q) missense variant in conjunction with c.1148delC. One of these patients also had a variant in *CNGA3*. The c.1208G > A variant is classified as pathogenic but presumably mild, likely representing a hypomorphic allele since it has also been found homozygous in 24 controls out of 140,050 (gnomAD v.2.1.1). It is, however, also a frequently occurring variant in ACHM [36,48] and is found more frequently in patients than controls. The c.1148delC;c.1208G > A genotype has been described in patients with both ACHM and progressive retinal disease [37,49]. The two patients in our cohort with this genotype were both young (22 and 17 years old), and both had residual color vision, but both had worsening of BCVA during follow-up, which could be indicative of progressive disease. However, for one of these patients, worsening of BCVA was only seen in one (exotropic) eye. However, the possibility exists that patients with these variants have a mild phenotype of incomplete ACHM to begin with and thereafter a more progressive course of disease. More studies of patients with this genotype are needed to conclude this. An accessory *CNGA3* variant with the two *CNGB3* variants, one being c.1208G > A;p.(R403Q), was recently described as exacerbating CNG-channel retinopathy [48]. Our patient with this genotype had visual acuity better than 1.0 logMAR but was only 4 years old at the latest follow-up, so future follow-up is needed to establish if this patient has a more severe phenotype.

With respect to refractive error, patients with ACHM have classically been described as being predominately hyperopic [50], but we and others have found refractive error to be more broadly distributed [12,51]. The current study is one of the first to systematically document refraction with variants in different causative genes, and we found indications of possible differences in refractive error with variants in different genes, as also reported by other studies [12]. However, these findings were not statistically significant when comparing the distribution of refractive error pairwise for the majority of genes. A inherent limitation of our study is the rarity of ACHM and thereby the limited number of patients, especially with variants in *GNAT2*, *PDE6C*, and *PDE6H*. The small sample sizes makes it difficult to draw strong conclusions on possible genotype-phenotype correlations. Detecting significant differences in refractive outcomes across various groups based on causative genes may necessitate larger sample sizes. Additionally, the multiple statistical testing required for small sample sizes could also pose issues. Future studies systemically documenting refractive error with variants in different genes in different populations could be useful, not only to further explore a possible genotype-phenotype association, but if such an association does exist, it could also provide insights into the complex and multifactorial process of emmetropization.

With the development of new treatments for ACHM, natural history studies such as the present one are relevant to establish which age groups are the best candidates for treatment. In case of progressive disease, younger patients may benefit more than older patients. In this current study, progressive disease was concluded from a worsening of BCVA, both because it is a clinically meaningful parameter for patients and because OCT data were not consistently available due to the retrospective nature of the study. Other studies have found that structural measures, such as OCT staging, do not necessarily correlate well with more functional measures, such as BCVA, in ACHM [6,10]. Differences in structural changes, however, may still be relevant in predicting treatment response. 

The retrospective nature of the current study allows for a large cohort of patients to be studied with a longer follow-up period compared to other studies. However, it also has limitations, namely the variable follow-up time, the lack of OCT to investigate morphological changes and the inconsistency in the methods used to evaluate color vision and fundoscopic changes. Full-field ERG was used to establish the diagnosis, but since it was performed years ago, in some cases before the development of ISCEV standards, it could not be evaluated systematically. 

In conclusion, the current study describes the genetic background and phenotypical characteristics of Danish patients with ACHM. We present data from a large cohort, and the retrospective nature allows for a long follow up. In concordance with other studies, we found genotype-phenotype correlations in the severity of the condition to be sparse, but evidence presented here supports that progressive disease is more frequently occurring with variants in *CNGB3* and *PDE6C*. A mild phenotype was more commonly seen with variants in *GNAT2* and *PDE6H*. We do, however, also describe a patient with complete ACHM and variants in *PDE6H*, thereby broadening the phenotypic spectrum for this gene. We also found indications of differences in refractive error with variants in the different genes associated with ACHM, which could also provide potential insights into the process of emmetropization. To further explore this possibility, it would be beneficial for future studies on the phenotypic characteristics of ACHM to also report refractive error systematically.

With the development of new gene therapeutic treatments for ACHM, natural history studies with long follow-ups are important. The results presented here support the evidence of ACHM being a predominantly stable condition with respect to BCVA, which is highly relevant to patients. However, future large, prospective studies enabling the study of other factors, such as work using high-resolution imaging, are also important in establishing possible treatment response and timing of treatment.

## Figures and Tables

**Table 1:** Summary of clinical findings in all ACHM patients and in patients distributed according to specific causal genes.

	BCVA Right Eye Median LogMAR (IQR)	Residual Color Vision	Incomplete ACHM	Congenital Nystagmus	Visual Field Constriction	Central Atrophy	BCVA Decline during Follow-Up **	Age Range in Years	Follow-Up Range in Years
All genes	1.00 (0.20)	14% (8/57) *	11% (5/57) *	89% (72/81) *	5% (3/66) *	19% (14/73) *	12% (7/58) *	2–76	0–71
*CNGA3*	0.90 (0.20)	18% (2/11) *	13% (2/16) *	87% (13/15) *	9% (1/11) *	29% (4/14) *	10% (1/10) *	3–71	0–54
*CNGB3*	1.00 (0.11)	9% (3/32) *	6% (1/32) *	90% (47/52) *	5% (2/42) *	20% (9/46) *	11% (4/37) *	2–76	0–71
*GNAT2*	1.00 (0.07)	25% (1/4) *	25% (1/4) *	100% (4/4) *	0% (0/4) *	0% (0/4) *	0% (0/4) *	37–57	25–41
*PDE6C*	1.00 (0.00)	20% (1/5) *	0% (0/5) *	100% (5/5) *	0% (0/5) *	20% (1/5) *	50% (2/4) *	19–51	14–41
*PDE6H*	0.74 (0.26)	50% (1/2) *	100% (1/1) *	60% (3/5) *	0% (0/4) *	0% (0/4) *	0% (0/3) *	2–20	1–9

* Number of patients/patients with available data. For incomplete ACHM, available data are both BCVA and color vision. ** Reduction in visual acuity >0.2 logMAR from first to last follow-up, cause not specified.

## Data Availability

Data are available from the corresponding author on reasonable request, given that the sharing complies with GDPR rules.

## Supplementary Materials

The following supporting information can be downloaded at: https://www.mdpi.com/article/10.3390/genes14030690/s1: Table S1: Genotypes of the Danish ACHM patients, variant ACMG classification, references of previously published variants, and if the patient has been included in previously published genetic studies.
