# Supplementary material for: Genetic and Clinical Characterization of Danish Achromatopsia Patients
Source: Genes (Basel). 2023 Mar 10;14(3):690. doi: 10.3390/genes14030690 (PMC10048638; doi:10.3390/genes14030690)
Supplement: Supplementary file 1 [file genes-14-00690-s001.zip › genes-2245142-supplementary.pdf]

Supplementary table S1. Genotypes of the Danish ACHM patients, variant ACMG classification, references of previously published variants, and if the patient has been included in previously published genetic studies.

Patient ID	Family ID	Consanguinity	Gene	Variant	Protein	ACMG classification	gnomAD	Reference variant	Patient previously published?
7*	219	No	*CNGA3*	c.947G>A	p.W316*	Pathogenic (PVS1_strong, PM3_strong, PM2_sup, PS4_sup)	NP	[1]	No
			CNGA3	c.1495C>T	p.R499*	Pathogenic (PVS1_strong, PM3_strong, PM2_sup, PS4_moderate)	3/282291/0	[2]	
40	219	No	*CNGA3*	c.947G>A	p.W316*	Pathogenic (PVS1_strong, PM3_strong, PM2_sup, PS4_sup)	NP	[1]	No
			CNGA3	c.1495C>T	p.R499*	Pathogenic (PVS1_strong, PM3_strong, PM2_sup, PS4_moderate)	3/282291/0	[2]	
64	219	No	*CNGA3*	c.947G>A	p.W316*	Pathogenic (PVS1_strong, PM3_strong, PM2_sup, PS4_sup)	NP	[1]	No
			CNGA3	c.1495C>T	p.R499*	Pathogenic (PVS1_strong, PM3_strong, PM2_sup, PS4_moderate)	3/282291/0	[2]	
41	214	Yes	*CNGA3*	c.847C>T	p.R283W	Likely pathogenic (PM2_sup, PM5, PS4, PM3)	25/251293/0	[3]	Yes [1] (CHRO127)
			CNGA3	c.847C>T	p.R283W	Likely pathogenic (PM2_sup, PM5, PS4, PM3)	25/251293/0	[3]	
63*	214	Yes	*CNGA3*	c.847C>T	p.R283W	Likely pathogenic (PM2_sup, PM5, PS4, PM3)	25/251293/0	[3]	No
			CNGA3	c.847C>T	p.R283W	Likely pathogenic (PM2_sup, PM5, PS4, PM3)	25/251293/0	[3]	

42	209	No	*CNGA3*	c.1574G>A	p.G525D	Likely pathogenic (PP3_strong, PS3_sup, PS4_sup, PM2_sup, PM3_sup)	NP	[1,4]	No
			CNGA3	c.1694C>T	p.T565M	Likely pathogenic (PM2, PS4, PM3, PS3_MOD)	47/282635/0	[1]	
43	209	No	*CNGA3*	c.1574G>A	p.G525D	Likely pathogenic (PP3_strong, PS3_sup, PS4_sup, PM2_sup, PM3_sup)	NP	[1,4]	Yes [1] (CHRO79)
			CNGA3	c.1694C>T	p.T565M	Likely pathogenic (PM2, PS4, PM3, PS3_MOD)	47/282635/0	[1]	
96**	209	No	*CNGA3*	c.1574G>A	p.G525D	Likely pathogenic (PP3_strong, PS3_sup, PS4_sup, PM2_sup, PM3_sup)	NP	[1,4]	No
			CNGA3	c.1694C>T	p.T565M	Likely pathogenic (PM2, PS4, PM3, PS3_MOD)	47/282635/0	[1]	
44		Yes	*CNGA3*	c.387delA	p.R131fs	Pathogenic (PVS1, PM3, PM2_sup, PS4_sup)	NP	[5]	No
			CNGA3	c.387delA	p.R131fs	Pathogenic (PVS1, PM3, PM2_sup, PS4_sup)	NP	[5]	
45		Yes	*CNGA3*	c.1641C>A	p.F547L	Pathogenic (PS4, PM3_strong, PM2_sup, PS3_mod, PP3_moderate)	43/282657/1	[1]	No
			CNGA3	c.1641C>A	p.F547L	Pathogenic (PS4, PM3_strong, PM2_sup, PS3_mod, PP3_moderate)	43/282657/1	[1]	
46	203	No	*CNGA3*	c.847C>T	p.R283W	Pathogenic (PS4_strong, PM3_very strong, PM2_sup, PP3_moderate)	25/251293/0	[3]	Yes [1] (CHRO74)
			CNGA3	c.1688G>A	p.R563H	Pathogenic (PS4, PM3_strong, PP3_strong, PM2_sup)	20/282622/0	[1]	
62*	203	No	*CNGA3*	c.847C>T	p.R283W	Pathogenic (PS4_strong, PM3_strong, PM2_sup, PP3_moderate)	25/251293/0	[1]	No
			CNGA3	c.1688G>A	p.R563H	Pathogenic (PS4, PM3_very strong, PP3_strong, PM2_sup)	20/282622/0	[1]	

61		No	*CNGA3*	c.847C>T	p.R283W	Pathogenic (PS4_strong, PM3_very strong, PM2_sup, PP3_moderate)	25/251293/0	[1]	No
			CNGA3	c.1574G>A	p.G525D	Likely pathogenic (PP3_strong, PS3_sup, PS4_sup, PM2_sup, PM3_sup)	NP	[1]	
76		No	*CNGA3*	c.67C>T	p.R23*	Pathogenic (PVS1, PS4_moderate, PM2_sup)	10/282444/0	[6]	No
			CNGA3	c.130_151dup	p.A51fs	Pathogenic (PVS1, PM2_sup, PS4_sup, PM3_sup)	NP	[7]	
77	555	No	*CNGA3*	c.829C>T	p.R277C	Pathogenic (PS4, PM3_strong, PP3_strong, PS3_moderate, PM1, PM5, PM2_sup)	24/251326/0	[1]	No
			CNGA3	c.847C>T	p.R283W	Pathogenic (PS4_strong, PM3_very strong, PM2_sup, PP3_moderate)	25/251293/0	[1]	
78	555	No	*CNGA3*	c.829C>T	p.R277C	Pathogenic (PS4, PM3_strong, PP3_strong, PS3_moderate, PM1, PM5, PM2_sup)	24/251326/0	[1]	No
			CNGA3	c.847C>T	p.R283W	Pathogenic (PS4_strong, PM3_very strong, PM2_sup, PP3_moderate)	25/251293/0	[1]	
93		Unknown	*CNGA3*	c.1641C>A	p.F547L	Pathogenic (PS4, PM3_strong, PM2_sup, PS3_mod, PP3_moderate)	43/282657/1	[1]	No
			CNGA3	c.1641C>A	p.F547L	Pathogenic (PS4, PM3_strong, PM2_sup, PS3_mod, PP3_moderate)	43/282657/1	[1]	
92		No	*CNGB3*	c.1148delC	p.T383fs	Pathogenic (PVS1, PS4, PM3_strong, PM2_sup)	40/282682/0	[8]	No
			CNGB3	c.1208G>A	p.R403Q	Pathogenic (PS4_strong, PM3_very strong, PS3_mod, BS)	1128/281228/24	[9]	
			CNGA3	c.1669G>A	p.G557R	Pathogenic (PS4, PP3_strong, PM2_sup, PS3_sup)	40/282682/0	[3]	
1		No	*CNGB3*	c.1148delC	p.T383fs	Pathogenic (PVS1, PS4, PM3_strong, PM2_sup)	489/281215/2	[8]	No

			CNGB3	c.1148delC	p.T383fs	Pathogenic (PVS1, PS4, PM3_strong, PM2_sup)	489/281215/2	[8]	
6		No	*CNGB3*	c.1148delC	p.T383fs	Pathogenic (PVS1, PS4, PM3_strong, PM2_sup)	489/281215/2	[8]	No
			CNGB3	c.1148delC	p.T383fs	Pathogenic (PVS1, PS4, PM3_strong, PM2_sup)	489/281215/2	[8]	
9		No	*CNGB3*	c.1148delC	p.T383fs	Pathogenic (PVS1, PS4, PM3_strong, PM2_sup)	489/281215/2	[8]	No
			CNGB3	c.1148delC	p.T383fs	Pathogenic (PVS1, PS4, PM3_strong, PM2_sup)	489/281215/2	[8]	
11		No	*CNGB3*	c.1148delC	p.T383fs	Pathogenic (PVS1, PS4, PM3_strong, PM2_sup)	489/281215/2	[8]	No
			CNGB3	c.1148delC	p.T383fs	Pathogenic (PVS1, PS4, PM3_strong, PM2_sup)	489/281215/2	[8]	
12		No	*CNGB3*	c.1148delC	p.T383fs	Pathogenic (PVS1, PS4, PM3_strong, PM2_sup)	489/281215/2	[8]	No
			CNGB3	c.1148delC	p.T383fs	Pathogenic (PVS1, PS4, PM3_strong, PM2_sup)	489/281215/2	[8]	
10	201	Yes	*CNGB3*	c.1148delC	p.T383fs	Pathogenic (PVS1, PS4, PM3_strong, PM2_sup)	489/281215/2	[8]	No
			CNGB3	c.1148delC	p.T383fs	Pathogenic (PVS1, PS4, PM3_strong, PM2_sup)	489/281215/2	[8]	
21	201	Yes	*CNGB3*	c.1148delC	p.T383fs	Pathogenic (PVS1, PS4, PM3_strong, PM2_sup)	489/281215/2	[8]	No
			CNGB3	c.1148delC	p.T383fs	Pathogenic (PVS1, PS4, PM3_strong, PM2_sup)	489/281215/2	[8]	
13	212	No	*CNGB3*	c.1148delC	p.T383fs	Pathogenic (PVS1, PS4, PM3_strong, PM2_sup)	489/281215/2	[8]	No
			CNGB3	c.1148delC	p.T383fs	Pathogenic (PVS1, PS4, PM3_strong, PM2_sup)	489/281215/2	[8]	
60*	212	No	*CNGB3*	c.1148delC	p.T383fs	Pathogenic (PVS1, PS4, PM3_strong, PM2_sup)	489/281215/2	[8]	No
			CNGB3	c.1148delC	p.T383fs	Pathogenic (PVS1, PS4, PM3_strong, PM2_sup)	489/281215/2	[8]	
14		No	*CNGB3*	c.1148delC	p.T383fs	Pathogenic (PVS1, PS4, PM3_strong, PM2_sup)	489/281215/2	[8]	No

			CNGB3	c.1148delC	p.T383fs	Pathogenic (PVS1, PS4, PM3_strong, PM2_sup)	489/281215/2	[8]	
19		No	*CNGB3*	c.1148delC	p.T383fs	Pathogenic (PVS1, PS4, PM3_strong, PM2_sup)	489/281215/2	[8]	No
			CNGB3	c.1148delC	p.T383fs	Pathogenic (PVS1, PS4, PM3_strong, PM2_sup)	489/281215/2	[8]	
15*	205	No	*CNGB3*	c.1148delC	p.T383fs	Pathogenic (PVS1, PS4, PM3_strong, PM2_sup)	489/281215/2	[8]	Yes [10] (clinical findings)
			CNGB3	c.1148delC	p.T383fs	Pathogenic (PVS1, PS4, PM3_strong, PM2_sup)	489/281215/2	[8]	
20	205	No	*CNGB3*	c.1148delC	p.T383fs	Pathogenic (PVS1, PS4, PM3_strong, PM2_sup)	489/281215/2	[8]	Yes [10] (clinical findings)
			CNGB3	c.1148delC	p.T383fs	Pathogenic (PVS1, PS4, PM3_strong, PM2_sup)	489/281215/2	[8]	
94*	205	No	*CNGB3*	c.1148delC	p.T383fs	Pathogenic (PVS1, PS4, PM3_strong, PM2_sup)	489/281215/2	[8]	Yes [10] (clinical findings)
			CNGB3	c.1148delC	p.T383fs	Pathogenic (PVS1, PS4, PM3_strong, PM2_sup)	489/281215/2	[8]	
27*	205	No	*CNGB3*	c.1148delC	p.T383fs	Pathogenic (PVS1, PS4, PM3_strong, PM2_sup)	489/281215/2	[8]	Yes [10] (clinical findings)
			CNGB3	c.1148delC	p.T383fs	Pathogenic (PVS1, PS4, PM3_strong, PM2_sup)	489/281215/2	[8]	
16		No	*CNGB3*	c.1148delC	p.T383fs	Pathogenic (PVS1, PS4, PM3_strong, PM2_sup)	489/281215/2	[8]	No
			CNGB3	c.1148delC	p.T383fs	Pathogenic (PVS1, PS4, PM3_strong, PM2_sup)	489/281215/2	[8]	
17		No	*CNGB3*	c.1148delC	p.T383fs	Pathogenic (PVS1, PS4, PM3_strong, PM2_sup)	489/281215/2	[8]	No
			CNGB3	c.1148delC	p.T383fs	Pathogenic (PVS1, PS4, PM3_strong, PM2_sup)	489/281215/2	[8]	
18		No	*CNGB3*	c.1148delC	p.T383fs	Pathogenic (PVS1, PS4, PM3_strong, PM2_sup)	489/281215/2	[8]	No
			CNGB3	c.1148delC	p.T383fs	Pathogenic (PVS1, PS4, PM3_strong, PM2_sup)	489/281215/2	[8]	
22		No	*CNGB3*	c.1148delC	p.T383fs	Pathogenic (PVS1, PS4, PM3_strong, PM2_sup)	489/281215/2	[8]	No

			CNGB3	c.1148delC	p.T383fs	Pathogenic (PVS1, PS4, PM3_strong, PM2_sup)	489/281215/2	[8]	
23		No	*CNGB3*	c.1148delC	p.T383fs	Pathogenic (PVS1, PS4, PM3_strong, PM2_sup)	489/281215/2	[8]	No
			CNGB3	c.1148delC	p.T383fs	Pathogenic (PVS1, PS4, PM3_strong, PM2_sup)	489/281215/2	[8]	
24		No	*CNGB3*	c.1148delC	p.T383fs	Pathogenic (PVS1, PS4, PM3_strong, PM2_sup)	489/281215/2	[8]	No
			CNGB3	c.1148delC	p.T383fs	Pathogenic (PVS1, PS4, PM3_strong, PM2_sup)	489/281215/2	[8]	
25		No	*CNGB3*	c.1148delC	p.T383fs	Pathogenic (PVS1, PS4, PM3_strong, PM2_sup)	489/281215/2	[8]	No
			CNGB3	c.1148delC	p.T383fs	Pathogenic (PVS1, PS4, PM3_strong, PM2_sup)	489/281215/2	[8]	
26		No	*CNGB3*	c.1148delC	p.T383fs	Pathogenic (PVS1, PS4, PM3_strong, PM2_sup)	489/281215/2	[8]	No
			CNGB3	c.1148delC	p.T383fs	Pathogenic (PVS1, PS4, PM3_strong, PM2_sup)	489/281215/2	[8]	
28*	204	No	*CNGB3*	c.1148delC	p.T383fs	Pathogenic (PVS1, PS4, PM3_strong, PM2_sup)	489/281215/2	[8]	No
			CNGB3	c.1148delC	p.T383fs	Pathogenic (PVS1, PS4, PM3_strong, PM2_sup)	489/281215/2	[8]	
29*	204	No	*CNGB3*	c.1148delC	p.T383fs	Pathogenic (PVS1, PS4, PM3_strong, PM2_sup)	489/281215/2	[8]	No
			CNGB3	c.1148delC	p.T383fs	Pathogenic (PVS1, PS4, PM3_strong, PM2_sup)	489/281215/2	[8]	
30	204	No	*CNGB3*	c.1148delC	p.T383fs	Pathogenic (PVS1, PS4, PM3_strong, PM2_sup)	489/281215/2	[8]	No
			CNGB3	c.1148delC	p.T383fs	Pathogenic (PVS1, PS4, PM3_strong, PM2_sup)	489/281215/2	[8]	
95**	204	No	*CNGB3*	c.1148delC	p.T383fs	Pathogenic (PVS1, PS4, PM3_strong, PM2_sup)	489/281215/2	[8]	No
			CNGB3	c.1148delC	p.T383fs	Pathogenic (PVS1, PS4, PM3_strong, PM2_sup)	489/281215/2	[8]	
31		Yes	*CNGB3*	c.1148delC	p.T383fs	Pathogenic (PVS1, PS4, PM3_strong, PM2_sup)	489/281215/2	[8]	Yes [11] (clinical findings)

			CNGB3	c.1148delC	p.T383fs	Pathogenic (PVS1, PS4, PM3_strong, PM2_sup)	489/281215/2	[8]	
32		No	*CNGB3*	c.1148delC	p.T383fs	Pathogenic (PVS1, PS4, PM3_strong, PM2_sup)	489/281215/2	[8]	No
			CNGB3	c.1148delC	p.T383fs	Pathogenic (PVS1, PS4, PM3_strong, PM2_sup)	489/281215/2	[8]	
33		No	*CNGB3*	c.1148delC	p.T383fs	Pathogenic (PVS1, PS4, PM3_strong, PM2_sup)	489/281215/2	[8]	No
			CNGB3	c.1148delC	p.T383fs	Pathogenic (PVS1, PS4, PM3_strong, PM2_sup)	489/281215/2	[8]	
34		No	*CNGB3*	c.1148delC	p.T383fs	Pathogenic (PVS1, PS4, PM3_strong, PM2_sup)	489/281215/2	[8]	No
			CNGB3	c.1148delC	p.T383fs	Pathogenic (PVS1, PS4, PM3_strong, PM2_sup)	489/281215/2	[8]	
47		No	*CNGB3*	c.1148delC	p.T383fs	Pathogenic (PVS1, PS4, PM3_strong, PM2_sup)	489/281215/2	[8]	No
			CNGB3	c.1148delC	p.T383fs	Pathogenic (PVS1, PS4, PM3_strong, PM2_sup)	489/281215/2	[8]	
48	1089	Yes	*CNGB3*	c.1148delC	p.T383fs	Pathogenic (PVS1, PS4, PM3_strong, PM2_sup)	489/281215/2	[8]	No
			CNGB3	c.1148delC	p.T383fs	Pathogenic (PVS1, PS4, PM3_strong, PM2_sup)	489/281215/2	[8]	
50*	1089	Yes	*CNGB3*	c.1148delC	p.T383fs	Pathogenic (PVS1, PS4, PM3_strong, PM2_sup)	489/281215/2	[8]	No
			CNGB3	c.1148delC	p.T383fs	Pathogenic (PVS1, PS4, PM3_strong, PM2_sup)	489/281215/2	[8]	
59		No	*CNGB3*	c.1148delC	p.T383fs	Pathogenic (PVS1, PS4, PM3_strong, PM2_sup)	489/281215/2	[8]	No
			CNGB3	c.1148delC	p.T383fs	Pathogenic (PVS1, PS4, PM3_strong, PM2_sup)	489/281215/2	[8]	
97**		Unknown	*CNGB3*	c.1148delC	p.T383fs	Pathogenic (PVS1, PS4, PM3_strong, PM2_sup)	489/281215/2	[8]	No
			CNGB3	c.1148delC	p.T383fs	Pathogenic (PVS1, PS4, PM3_strong, PM2_sup)	489/281215/2	[8]	
98		No	*CNGB3*	c.1148delC	p.T383fs	Pathogenic (PVS1, PS4, PM3_strong, PM2_sup)	489/281215/2	[8]	No

			CNGB3	c.1148delC	p.T383fs	Pathogenic (PVS1, PS4, PM3_strong, PM2_sup)	489/281215/2	[8]	
2	217	No	*CNGB3*	c.1148delC	p.T383fs	Pathogenic (PVS1, PS4, PM3_strong, PM2_sup)	489/281215/2	[8]	No
			CNGB3	c.1432C>T	p.R478*	Pathogenic (PVS1, PS4, PM2_sup), PM3	5/251095/0	[12]	
37*	217	No	*CNGB3*	c.1148delC	p.T383fs	Pathogenic (PVS1, PS4, PM3_strong, PM2_sup)	489/281215/2	[8]	No
			CNGB3	c.1432C>T	p.R478*	Pathogenic (PVS1, PS4, PM2_sup), PM3	5/251095/0	[12]	
38*	217	No	*CNGB3*	c.1148delC	p.T383fs	Pathogenic (PVS1, PS4, PM3_strong, PM2_sup)	489/281215/2	[8]	No
			CNGB3	c.1432C>T	p.R478*	Pathogenic (PVS1, PS4, PM2_sup) PM3	5/251095/0	[12]	
4*	210	No	*CNGB3*	c.1148delC	p.T383fs	Pathogenic (PVS1, PS4, PM3_strong, PM2_sup)	489/281215/2	[8]	No
			CNGB3	c.467C>T	p.S156F	VUS (PS4_mod, PM2_sup, BP4)	14/282812/0	[12]	
39	210	No	*CNGB3*	c.1148delC	p.T383fs	Pathogenic (PVS1, PS4, PM3_strong, PM2_sup)	489/281215/2	[8]	No
			CNGB3	c.467C>T	p.S156F	VUS (PS4_mod, PM2_sup, BP4)	14/282812/0	[12]	
8		No	*CNGB3*	c.1148delC	p.T383fs	Pathogenic (PVS1, PS4, PM3_strong, PM2_sup)	489/281215/2	[8]	No
			CNGB3	c.1299_1300delGT	p.F434fs	Pathogenic (PVS1, PS4_sup, PM2_sup)	NP	[13]	
35	207	No	*CNGB3*	c.1148delC	p.T383fs	Pathogenic (PVS1, PS4, PM3_strong, PM2_sup)	489/281215/2	[8]	No
			CNGB3	c.886-896del11insT	p.T296fs	Pathogenic (PVS1, PS4_mod, PM2_sup)	NP	[13]	
36*	207	No	*CNGB3*	c.1148delC	p.T383fs	Pathogenic (PVS1, PS4, PM3_strong, PM2_sup)	489/281215/2	[8]	No
			CNGB3	c.886-896del11insT	p.T296fs	Pathogenic (PVS1, PS4_mod, PM2_sup)	NP	[13]	
51		No	*CNGB3*	c.1148delC	p.T383fs	Pathogenic (PVS1, PS4, PM3_strong, PM2_sup)	489/281215/2	[8]	No
			CNGB3	c.589_590delTT	p.L197fs	Pathogenic (PVS1, PS4_sup, PM2_sup)	NP	[13]	

69		No	*CNGB3*	c.1148delC	p.T383fs	Pathogenic (PVS1, PS4, PM3_strong, PM2_sup)	489/281215/2	[8]	No
			CNGB3	c.1431delG	p.K477fs	Pathogenic (PVS1, PM2_sup, PM3)	NP	Novel	
74		No	*CNGB3*	c.1148delC	p.T383fs	Pathogenic (PVS1, PS4, PM3_strong, PM2_sup)	489/281215/2	[8]	No
			CNGB3	c.1700G>A	p.G567E	Likely pathogenic (PS4_sup, PP3_strong, PM2_sup)	1/251083/0	[14]	
80		No	*CNGB3*	c.1148delC	p.T383fs	Pathogenic (PVS1, PS4, PM3_strong, PM2_sup)	489/281215/2	[8]	No
			CNGB3	c.1208G>A	p.R403Q	Pathogenic (PS4_strong, PM3_very strong, PS3_mod, BS)	1128/281228/24	[9]	
100		No	*CNGB3*	c.1148delC	p.T383fs	Pathogenic (PVS1, PS4, PM3_strong, PM2_sup)	489/281215/2	[8]	No
			CNGB3	c.1208G>A	p.R403Q	Pathogenic (PS4_strong, PM3_very strong, PS3_mod, BS)	1128/281228/24	[9]	
52	203	Yes	*CNGB3*	c.1430_1431delinsC	p.K477fs*17	Pathogenic (PVS1, PM2_sup, PM3)	NP	[13]	Yes [15–17] (CHRO89)
			CNGB3	c.1430_1431delinsC	p.K477fs*17	Pathogenic (PVS1, PM2_sup, PM3)	NP	[13]	
54	203	Yes	*CNGB3*	c.1430_1431delinsC	p.K477fs*17	Pathogenic (PVS1, PM2_sup, PM3)	NP	[13]	Yes [15–17] (CHRO89)
			CNGB3	c.1430_1431delinsC	p.K477fs*17	Pathogenic (PVS1, PM2_sup, PM3)	NP	[13]	
55	215	Yes	*CNGB3*	c.702T>A	p.C234*	Pathogenic (PVS1, PM2_sup, PM3)	NP	[13]	No
			CNGB3	c.702T>A	p.C234*	Pathogenic (PVS1, PM2_sup, PM3)	NP	[13]	
56*	215	Yes	*CNGB3*	c.702T>A	p.C234*	Pathogenic (PVS1, PM2_sup, PM3)	NP	[13]	No
			CNGB3	c.702T>A	p.C234*	Pathogenic (PVS1, PM2_sup, PM3)	NP	[13]	
72		No	*CNGB3*	c.1700G>A	p.G567E	Likely pathogenic PP3_strong, PM2_sup, PM3_sup, PS4_sup)	1/251083/0	[14]	No
			CNGB3	c.1700G>A	p.G567E	Likely pathogenic PP3_strong, PM2_sup, PM3_sup, PS4_sup)	1/251083/0	[14]	

75		Yes	*CNGB3*	arr[Hg19]8q21.3 (87,736,031-87,742,065=x0 hmz mat pat	Del exon 3				No
			CNGB3	arr[Hg19]8q21.3 (87,736,031-87,742,065=x0 hmz mat pat	Del exon 3				
99		No	*CNGB3*	c.702T>A	p.C234*	Pathogenic (PVS1, PM2_sup, PM3)	NP	[13]	No
			CNGB3	c.756C>G	p.Y252*	Pathogenic (PVS1, PM2_sup, PS4_sup)	1/251445/0	[13]	
57	202	Yes	*GNAT2*	c.481C>T	p.R161*	Pathogenic (PVS1, PS4_mod, PM2_sup)	5/282855/0	[18]	Yes [18] (CHRO73)
			GNAT2	c.481C>T	p.R161*	Pathogenic (PVS1, PS4_mod, PM2_sup)	5/282855/0	[18]	
82	202	Yes	*GNAT2*	c.481C>T	p.R161*	Pathogenic (PVS1, PS4_mod, PM2_sup)	5/282855/0	[18]	Yes [18] (CHRO73)
			GNAT2	c.481C>T	p.R161*	Pathogenic (PVS1, PS4_mod, PM2_sup)	5/282855/0	[18]	
83	101 (ID79 cousin)	No	*GNAT2*	c.285-291delinsCTGTAT	p.A96fs	Likely pathogenic (PVS1, PM2_sup)	NP	[19]	Yes [18–20] (CHRO87)
			GNAT2	c.461+24G>A	p.?	Likely pathogenic (PS3, PM2_sup, PM3)	NP	[20]	
79	101	No	*GNAT2*	c.285-291delinsCTGTAT	p.A96fs	Likely pathogenic (PVS1, PM2_sup)	NP	[19]	Yes [18–20] (CHRO87)
			GNAT2	c.285-291delinsCTGTAT	p.A96fs	Likely pathogenic (PVS1, PM2_sup)	NP	[19]	
67		Yes	*PDE6C*	c.1936-2A>G	p.?	Pathogenic (PVS1, PM3_sup, PM2_sup)	NP	Novel	No
			PDE6C	c.1936-2A>G	p.?	Pathogenic (PVS1, PM3_sup, PM2_sup)	NP	Novel	

81		No	*PDE6C*	c.85C>T	p.R29W	Pathogenic (PS4, PS3, PM2_sup, PM3)	NP	[21]	Yes [22] (CHRO319)
			PDE6C	c.2144+1G>A	p.?	Likely pathogenic (PVS1_strong, PM2_sup; PS4_sup)	NP	[22]	
84	218	Yes	*PDE6C*	c.297C>G	p.F99L	VUS (PM2_sup)	NP	Novel	No
			PDE6C	c.939+5G>T	p.?	Likely pathogenic (PVS1_strong, PM5, PM2_sup)	NP	Novel	
85*	218	Yes	*PDE6C*	c.297C>G	p.F99L	VUS (PM2_sup)	NP	Novel	No
			PDE6C	c.939+5G>T	p.?	Likely pathogenic (PVS1_strong, PM5, PM2_sup)	NP	Novel	
91		Yes	*PDE6C*	c.1071+5G>A	p.?	Pathogenic (PVS1, PM2_sup, PM3_sup)	NP	Novel	No
			PDE6C	c.1071+5G>A	p.?	Pathogenic (PVS1, PM2_sup, PM3_sup)	NP	Novel	
86	423	Yes	*PDE6H*	c.35C>G	p.S12*	Pathogenic (PVS1, PS4, PM3, PM2_sup)	26/282590/0	[23]	No
			PDE6H	c.35C>G	p.S12*	Pathogenic (PVS1, PS4, PM3, PM2_sup)	26/282590/0	[23]	
87*	423	Yes	*PDE6H*	c.35C>G	p.S12*	Pathogenic (PVS1, PS4, PM3, PM2_sup)	26/282590/0	[23]	No
			PDE6H	c.35C>G	p.S12*	Pathogenic (PVS1, PS4, PM3, PM2_sup)	26/282590/0	[23]	
88	409	No	*PDE6H*	c.35C>G	p.S12*	Pathogenic (PVS1, PS4, PM3, PM2_sup)	26/282590/0	[23]	No
			PDE6H	c.35C>G	p.S12*	Pathogenic (PVS1, PS4, PM3, PM2_sup)	26/282590/0	[23]	
89	409	No	*PDE6H*	c.35C>G	p.S12*	Pathogenic (PVS1, PS4, PM3, PM2_sup)	26/282590/0	[23]	No
			PDE6H	c.35C>G	p.S12*	Pathogenic (PVS1, PS4, PM3, PM2_sup)	26/282590/0	[23]	
90	409	No	*PDE6H*	c.35C>G	p.S12*	Pathogenic (PVS1, PS4, PM3, PM2_sup)	26/282590/0	[23]	No
			PDE6H	c.35C>G	p.S12*	Pathogenic (PVS1, PS4, PM3, PM2_sup)	26/282590/0	[23]	

*Variant has been identified in sibling
**Clinical data not available

References

1. Wissinger, B.; Gamer, D.; Jägle, H.; Giorda, R.; Marx, T.; Mayer, S.; Tippmann, S.; Broghammer, M.; Jurklies, B.; Rosenberg, T.; et al. CNGA3 Mutations in Hereditary Cone Photoreceptor Disorders. *Am. J. Hum. Genet.* **2001**, *69*, 722–737, doi:10.1086/323613.
2. Burgueño-Montañés, C.; Colunga Cueva, M.; Costales Álvarez, C. Una Nueva Mutación En El Gen CNGA3 Causante de Acromatopsia Incompleta. *Arch. Soc. Esp. Oftalmol.* **2014**, *89*, 107–109, doi:10.1016/j.oftal.2012.07.019.
3. Kohl, S.; Marx, T.; Giddings, I.; Jägle, H.; Jacobson, S.G.; Apfelstedt-Sylla, E.; Zrenner, E.; Sharpe, L.T.; Wissinger, B. Total Colourblindness Is Caused by Mutations in the Gene Encoding the α-Subunit of the Cone Photoreceptor CGMP-Gated Cation Channel. *Nat. Genet.* **1998**, *19*, 257–259, doi:10.1038/935.
4. Muraki-Oda, S.; Toyoda, F.; Okada, A.; Tanabe, S.; Yamade, S.; Ueyama, H.; Matsuura, H.; Ohji, M. Functional Analysis of Rod Monochromacy-Associated Missense Mutations in the CNGA3 Subunit of the Cone Photoreceptor CGMP-Gated Channel. *Biochem. Biophys. Res. Commun.* **2007**, *362*, 88–93, doi:10.1016/j.bbrc.2007.07.152.
5. Solaki, M.; Baumann, B.; Reuter, P.; Andreasson, S.; Audo, I.; Ayuso, C.; Balousha, G.; Benedicenti, F.; Birch, D.; Bitoun, P.; et al. Comprehensive Variant Spectrum of the CNGA3 Gene in Patients Affected by Achromatopsia. *Hum. Mutat.* **2022**, doi:10.1002/humu.24371.
6. Johnson, S.; Michaelides, M.; Aligianis, I.A.; Ainsworth, J.R.; Mollon, J.D.; Maher, E.R.; Moore, A.T.; Hunt, D.M. Achromatopsia Caused by Novel Mutations in Both CNGA3 and CNGB3. *J. Med. Genet.* **2004**, *41*, e20, doi:10.1136/jmg.2003.011437.
7. Zelinger, L.; Cideciyan, A. V; Kohl, S.; Schwartz, S.B.; Rosenmann, A.; Eli, D.; Sumaroka, A.; Roman, A.J.; Luo, X.; Brown, C.; et al. Genetics and Disease Expression in the CNGA3 Form of Achromatopsia: Steps on the Path to Gene Therapy. *Ophthalmology* **2015**, *122*, 997–1007, doi:10.1016/j.ophtha.2014.11.025.
8. Kohl, S.; Baumann, B.; Broghammer, M.; Jägle, H.; Sieving, P.; Kellner, U.; Spegal, R.; Anastasi, M.; Zrenner, E.; Sharpe, L.T.; et al. Mutations in the CNGB3 Gene Encoding the Beta-Subunit of the Cone Photoreceptor CGMP-Gated Channel Are Responsible for Achromatopsia (ACHM3) Linked to Chromosome 8q21. *Hum. Mol. Genet.* **2000**, *9*, 2107–2116, doi:10.1093/hmg/9.14.2107.
9. Michaelides, M.; Aligianis, I.A.; Ainsworth, J.R.; Good, P.; Mollon, J.D.; Maher, E.R.; Moore, A.T.; Hunt, D.M. Progressive Cone Dystrophy Associated with Mutation in CNGB3. *Invest. Ophthalmol. Vis. Sci.* **2004**, *45*, 1975–1982, doi:10.1167/iovs.03-0898.
10. S. Ry Andersen On Congenital Total Colou Blindness Coexisting with Heredo-Labyrinthine Deafness. *Acta Ophthalmol.* **1946**, *24*, 99–112.
11. Ejler Holm, C; Lodberg, C.V. A Family with Total Colour-Blindness. *Acta Ophthalmol.* **1940**, *18*, 224–258.
12. Kohl, S.; Varsanyi, B.; Antunes, G.A.; Baumann, B.; Hoyng, C.B.; Jägle, H.; Rosenberg, T.; Kellner, U.; Lorenz, B.; Salati, R.; et al. CNGB3 Mutations Account for 50% of All Cases with Autosomal Recessive Achromatopsia. *Eur. J. Hum. Genet.* **2005**, *13*, 302–308, doi:10.1038/sj.ejhg.5201269.
13. Mayer, A.K.; Cauwenbergh, C.; Rother, C.; Baumann, B.; Reuter, P.; Baere, E.; Wissinger, B.; Kohl, S. CNGB3 Mutation Spectrum Including Copy Number Variations in 552 Achromatopsia Patients. *Hum. Mutat.* **2017**, *38*, 1579–1591, doi:10.1002/humu.23311.
14. Jespersgaard, C.; Fang, M.; Bertelsen, M.; Dang, X.; Jensen, H.; Chen, Y.; Bech, N.; Dai, L.; Rosenberg, T.; Zhang, J.; et al. Molecular Genetic Analysis Using Targeted NGS Analysis of 677 Individuals with Retinal Dystrophy. *Sci. Rep.* **2019**, *9*, 1219, doi:10.1038/s41598-018-38007-2.
15. Weisschuh, N.; Mayer, A.K.; Strom, T.M.; Kohl, S.; Glöckle, N.; Schubach, M.; Andreasson, S.; Bernd, A.; Birch, D.G.; Hamel, C.P.; et al. Mutation Detection in Patients with Retinal Dystrophies Using Targeted Next Generation Sequencing. *PLoS One* **2016**, *11*, e0145951, doi:10.1371/journal.pone.0145951.
16. Rosenberg, Thomas; Olsen, Jens V.; Weissschuh, Nicole; Kohl, S.W.B. Old Mystery Solved: Achromatopsia, the Fuur Genealogy in Retrospective. *Ann. Case Reports* **2021**, *6*, 1–6, doi:10.29011/2574-7754.100628.
17. Franceschetti, A; Jaeger, W; Klein, D; Ohrt, V; Rickly, H. Etude Patho-Physiologique de La Grande Familie d'achromats de l'ile de Fur (Danemark). *Concil. Ophtalmol.* **1958**.
18. Felden, J.; Baumann, B.; Ali, M.; Audo, I.; Ayuso, C.; Bocquet, B.; Casteels, I.; Garcia-Sandoval, B.; Jacobson, S.G.; Jurklies, B.; et al. Mutation Spectrum and Clinical Investigation of Achromatopsia Patients with Mutations in the GNAT2 Gene. *Hum. Mutat.* **2019**, humu.23768, doi:10.1002/humu.23768.

19. Kohl, S.; Baumann, B.; Rosenberg, T.; Kellner, U.; Lorenz, B.; Vadalà, M.; Jacobson, S.G.; Wissinger, B. Mutations in the Cone Photoreceptor G-Protein α-Subunit Gene GNAT2 in Patients with Achromatopsia. *Am. J. Hum. Genet.* **2002**, *71*, 422–425, doi:10.1086/341835.
20. Rosenberg, T.; Baumann, B.; Kohl, S.; Zrenner, E.; Jorgensen, A.L.; Wissinger, B. Variant Phenotypes of Incomplete Achromatopsia in Two Cousins with GNAT2 Gene Mutations. *Investig. Opthalmology Vis. Sci.* **2004**, *45*, 4256, doi:10.1167/iovs.04-0317.
21. Thiadens, A.A.H.J.; den Hollander, A.I.; Roosing, S.; Nabuurs, S.B.; Zekveld-Vroon, R.C.; Collin, R.W.J.; De Baere, E.; Koenekoop, R.K.; van Schooneveld, M.J.; Strom, T.M.; et al. Homozygosity Mapping Reveals PDE6C Mutations in Patients with Early-Onset Cone Photoreceptor Disorders. *Am. J. Hum. Genet.* **2009**, *85*, 240–247, doi:10.1016/j.ajhg.2009.06.016.
22. Grau, T.; Artemyev, N.O.; Rosenberg, T.; Dollfus, H.; Haugen, O.H.; Cumhur Sener, E.; Jurklies, B.; Andreasson, S.; Kernstock, C.; Larsen, M.; et al. Decreased Catalytic Activity and Altered Activation Properties of PDE6C Mutants Associated with Autosomal Recessive Achromatopsia. *Hum. Mol. Genet.* **2011**, *20*, 719–730, doi:10.1093/hmg/ddq517.
23. Kohl, S.; Coppieters, F.; Meire, F.; Schaich, S.; Roosing, S.; Brennenstuhl, C.; Bolz, S.; van Genderen, M.M.; Riemslag, F.C.C.; Lukowski, R.; et al. A Nonsense Mutation in PDE6H Causes Autosomal-Recessive Incomplete Achromatopsia. *Am. J. Hum. Genet.* **2012**, *91*, 527–532, doi:10.1016/j.ajhg.2012.07.006.
